# Case–Control Study of *Clostridium innocuum* Infection, Taiwan

**DOI:** 10.3201/eid2803.204421

**Published:** 2022-03

**Authors:** Yi-Ching Chen, Yi-Chun Kuo, Mi-Chi Chen, Young-Da Zhang, Chyi-Liang Chen, Puo-Hsien Le, Cheng-Hsun Chiu

**Affiliations:** Chang Gung University, Taoyuan, Taiwan (Y.-C. Chen, Y.-C. Kuo, C.-H. Chiu);; Chang Gung Memorial Hospital, Taoyuan (Y.-C. Chen, M.-C. Chen, Y.-D. Zhang, C.-L. Chen, P.-H. Le, C.-H. Chiu)

**Keywords:** *Clostridium innocuum*, *Clostridioides difficile*, extraintestinal clostridial infection, vancomycin resistance, antimicrobial resistance, bacteria, enteric infections, Taiwan

## Abstract

Vancomycin-resistant *Clostridium innocuum* was recently identified as an etiologic agent for antibiotic-associated diarrhea in humans. We conducted a case–control study involving 152 *C. innocuum*-infected patients during 2014–2019 in Taiwan, using 304 cases of *Clostridioides difficile* infection (CDI) matched by diagnosis year, age (+2 years), and sex as controls. The baseline characteristics were similar between the 2 groups. *C. innocuum*–infected patients experienced more extraintestinal clostridial infection and gastrointestinal tract–related complications than did patients with CDI. The 30-day mortality rate among *C. innocuum*–infected patients was 14.5%, and the overall rate was 23.0%. Chronic kidney disease, solid tumor, intensive care unit admission, and shock status were 4 independent risk factors for death. *C. innocuum* identified from clinical specimens should be recognized as a pathogen requiring treatment, and because of its intrinsic vancomycin resistance, precise identification is necessary to guide appropriate and timely antimicrobial therapy.

*Clostridium* species are obligate anaerobic, endospore-forming bacilli that usually colonize in the gastrointestinal tracts of humans. Of the >200 species of *Clostridium*, >30 are potential pathogens in humans, such as *C. perfringens* and *Clostridioides difficile.* However, *C. innocuum* has rarely been described as associated with human disease. 

*C. innocuum* was first identified in the 1960s among 8 patients in the United States; the name, innocuum, described its lack of virulence (*1*,*2*). It was challenging to distinguish *C. innocuum* from other *Clostridium* species (especially *C. ramosum* and *C. clostridioforme*, together called the RIC group) because of their similar phenotypes of atypical clostridial colonial morphology, rare spore-forming features, and fatty acid pattern (*3*–*5*). Identifying *C. innocuum* has become faster and more accurate after the introduction of molecular techniques such as 16S RNA sequencing and matrix-associated laser desorption/ionization time-of-flight (MALDI-TOF) mass spectrometry (*6*). 

In 1995, Cutrona et al. reported the first case of endocarditis caused by *C. innocuum* (*7*). Although the bacterium was considered less pathogenic and seldom caused infections previously, more and more clinical evidence has emerged since 2000s, suggesting *C. innocuum* might be a potential cause of antibiotic-associated diarrhea and of extraintestinal clostridial infection (EICI), such as bacteremia, intra-abdominal infection, and endocarditis (*8*–*10*). However, we are not aware of a study of *C. innocuum* infection with a large enough cohort of patients to describe its clinical characteristics.

Precise diagnosis of *C. innocuum* is necessary because of its unique intrinsic resistance to vancomycin, presumably caused by the presence of 2 chromosomal genes that enable the synthesis of a peptidoglycan precursor terminating in serine with low vancomycin affinity (*9*,*11*). Although vancomycin is one of the recommended antimicrobial drugs to treat infections caused by *Clostridium* species, especially *C. difficile*, intrinsic resistance to vancomycin in *C. innocuum* poses the risk for inappropriate treatment for patients who acquire *C. innocuum* infection (*12*). *C. difficile* is one of the most representative clostridial species to cause human disease and has been well investigated. In the United States, ≈500,000 infections were identified annually, and 15,000–30,000 deaths were associated with *C. difficile* infection (CDI) (*12*–*14*)

In previous studies, we demonstrated *C. innocuum* as a potential invasive pathogen causing severe colitis and EICI in a small case series and proved its cellular toxicity in vitro (*8*,*9*). Herein, we conducted a retrospective case–control study to describe and evaluate the clinical characteristics and outcomes of infections caused by *C. innocuum*. To this end, we selected case-patients with CDI as the control group.

Institute Review Boards in Chang Gung Memorial Hospital (CGMH; Taoyuan, Taiwan) approved the study, allowing review of the medical data of the patients (IRB#201900906B0). A waiver of consent was granted given the retrospective nature of the project and anonymous analysis of the clinical information of patients.

## Methods

### Study Design, Clinical Setting, and Case Enrollment

We conducted a retrospective case–control study at CGMH during 2014–2019. CGMH is a tertiary medical center accommodating 3,700 patient beds. We selected *C. difficile* as the control to better illustrate the clinical features of *C. innocuum* infection. The case and control groups were assigned in a 1:2 ratio and matched in the diagnosed year, age +2 years, and sex.

We identified cases with *C. innocuum* and *C. difficile* infections using the rapid ID 32A system (bioMérieux, https://www.biomerieux.com) and MALDI-TOF mass spectrometry Biotyper (Bruker Daltonik GmbH, https://www.bruker.com) (*15*–*17*). MALDI-TOF mass spectrometry was introduced in 2009 in the clinical microbiology laboratory of CGMH, but *C. innocuum* was not reported routinely because it was considered a clinically insignificant microorganism. To trace the cases infected with *C. innocuum*, we reviewed the original reporting database from the MALDI-TOF mass spectrometry system directly and identified the samples reporting *C. innocuum.* Our definition of a microbiologically confirmed *C. innocuum* infection was that the original report from the MALDI-TOF mass spectrometry database revealed *C. innocuum* in the strongest 2 signals and had signal scores >2.00. We defined *C. difficile* infections by the same rationale.

We reviewed baseline information of each patient and enlisted all patients with *C. innocuum* infection in the study. We defined *C. difficile* infection as a positive PCR-based toxin assay with presence of clinical symptoms compatible with the infection, or a positive culture of *C. difficile* with compatible clinical symptoms (e.g., documented diarrhea or radiologic features of toxic megacolon). We excluded cases with concomitant *C. innocuum* and *C. difficile* isolated from the same clinical sample from the study. For the case-control matching, 3 authors (Y.-C. Chen, Y.-C. Kuo, and M.-C. Chen) reviewed baseline information of all cases with *C. innocuum* and *C. difficile* infection. We randomly selected 2 controls for each case, matched by diagnostic year, age (+2 years), and sex of the index case. If no controls were eligible from these 3 matching variables, then we dropped the sex criterium, followed by the age criterium if necessary. After the matching process, we further reviewed the clinical information of these patients.

### Clinical Data Resources, Variables, and Definition

We collected demographic data, clinical manifestations, laboratory testing results, images, and microbiology reports through an electronic medical record system (EMR). Demographic data were age, sex, race, underlying systemic diseases, and acquisition modality (community vs. hospital). We defined hospital-acquired infection as the symptoms that occurred >48 hours after admission, or <4 weeks after discharge from a healthcare facility; otherwise, it was classified as a community-acquired infection (*18*). We calculated the Charlson Comorbidity Index score for each patient to represent the baseline physiologic condition affected by underlying disease. The index is composed of 19 underlying conditions in 4 categories. Each category had a weighted score based on the risk for 1- and 10-year mortality rate (*19*).

We recorded clinical symptoms such as diarrhea, fever, bloody stool, abdominal pain, vomiting, and abdominal distension. We also reviewed disease-related complications, including toxic megacolon, ileus, bowel perforation, and shock. Recurrent infection was defined if the patient had a repeated microbiological culture from the same specimen source within 8 weeks of initial documented symptoms resolution (*20*,*21*). Outcome assessment included 30-day, 90-day, and overall deaths after the infection. We reviewed previous antibiotic exposures according to each class: penicillins, cephalosporins, carbapenems, fluoroquinolones, aminoglycosides, macrolides, tetracyclines, glycopeptides, oxazolids, polymyxins, lincosamides, and metronidazole. We defined antibiotic exposure rates as the percentage of patients who received any drugs <30 days before *C. innocuum* or *C. difficile* infection and duration of antibiotic exposure as total days of any antimicrobial drug use in a patient <30 days before the event of *C. innocuum* or *C. difficile* infection.

### Bacterial Isolation and Identification

We performed anaerobic bacterial cultures in the clinical microbiology laboratory, as described previously (*9*). We streaked all the anaerobic samples onto the selective agar plate, including CDC-ANA-BAP (anaerobic blood agar plate), CDC-ANA-PEA (anaerobic phenylethyl alcohol blood agar plate), and BBE/KVLB (Bacteroides bile esculin and laked kanamycin) bi-plate. We incubated agar plates in anaerobic conditions (90% N_2_/10% CO_2_) at 37°C for 5 days. We grossly reviewed the growing colonies on plate and analyzed 1 representative colony for each agar plate by the rapid ID 32A system (bioMérieux) for identification of the microorganisms.

### Antimicrobial Susceptibility Testing

We tested antimicrobial susceptibilities to clindamycin, metronidazole, penicillin, piperacillin, and ampicillin/sulbactam by the break-point agar dilution method according to Clinical and Laboratory Standards Institute criteria (document M11-A8) for anaerobic bacteria (*22*). We used interpretive criteria in document M100S to determine susceptibility (*22*).

### Statistical Analysis

We performed statistical analysis by SPSS Statistics 24.0 (SPSS Inc., https://www.ibm.com/products/spss-statistics). For continuous variables, we determined significance by using the independent t test or Mann-Whitney U test as appropriate. If the continuous variable had outliers and did not fit the normal distribution, variables were shown as median (interquartile range, range). We analyzed the categorical variables by χ^2^ test and considered p<0.05 statistically significant. We obtained odds ratios (ORs) from cross-tabulation and analyzed the p value of ORs by univariate logistic regression. We estimated mortality rate at 30 days and 90 days after the positive culture and analyzed by Kaplan-Meier survival analysis using methods described previously (*23*). In addition, we examined risk factors associated with 30- and 90-day mortality in both groups by logistic regression.

## Results

### Participants and Demographic Information

By the MALDI-TOF mass spectrometry system, 180 samples yielded the growth of *C. innocuum.* We excluded 22 of those from further analysis because of lack of access to clinical information and 6 because of concomitant isolation of *C. innocuum* and *C. difficile* from the same sample (CI group). We matched the control group with *C. innocuum* samples in accordance with the study criteria. From 1,134 *C. difficile* cases during the study period, we enrolled 304 cases as controls (CD group). All control cases were matched precisely on diagnostic year and age (+2 years); 25 controls were not matched on sex. The mean patient age for the 456 cases was 66.7 years, and 58.3% of patients were male (Table 1). Both groups were similar regarding age, sex, and Charlson Comorbidity Index score (5.7 + 3.2 for CI and 6.2 + 3.3 for CD). Subgroup analysis of each age group (<50, 50–60, 60–70, 70–80, and >80 years) also revealed no statistical difference. Overall, 8 pediatric patients were recruited, 3 in the CI group and 5 in the CD group. Regarding underlying systemic diseases, the CD group showed more patients with chronic kidney disease (18.4% vs. 30.9%; p = 0.005) (Table 1). Of note, more patients acquired the infection in the community in the CI group (33.6% vs. 16.8%; odds ratio [OR] 2.5, 95% CI 1.6–3.9; p<0.001) (Table 1).

**Table 1:** Baseline characteristics and clinical diagnoses in 2 groups of patients by the infecting *Clostridium* species in case–control study of *C. innocuum* infection, Taiwan*

Variable	Total, N = 456	*C. innocuum*, n = 152	*Clostridioides difficile*, n = 304	OR (95% CI)	p value
Age, mean (SD)†	66.7 (18.2)	66.6 (18.3)	66.7 (18.1)	NA	0.978
Sex					
M	266 (58.3)	97 (63.8)	169 (55.6)	1.41 (0.94‒2.10)	0.094
F	190 (41.7)	55 (36.2)	135 (44.4)	0.71 (0.48‒1.06)	0.094
Hospitalization	439 (96.2)	142 (93.4)	297 (97.7)	0.34 (0.13‒0.90)	0.03
No. days, median (IQR, range)‡	22 (36, 0‒492)	14 (33, 0‒492)	26 (36, 0‒409)	NA	<0.001
Charlson Comorbidity Index, mean (SD)†	6.1 (3.2)	5.7 (3.2)	6.2 (3.3)	NA	0.100
Diabetes mellitus	135 (29.6)	52 (34.2)	83 (27.3)	1.39 (0.91‒2.11)	0.128
Chronic kidney disease	122 (26.8)	28 (18.4)	94 (30.9)	0.50 (0.31‒0.81)	0.005
Congestive heart failure	45 (9.9)	12 (7.9)	33 (10.9)	0.70 (0.35‒1.40)	0.315
AIDS	4 (0.9)	1 (0.7)	3 (1.0)	0.66 (0.07‒6.44)	0.724
Solid tumor	138 (30.3)	42 (27.6)	96 (31.6)	0.83 (0.54‒1.27)	0.387
Initial ICU admission	65 (14.3)	36 (23.7)	29 (9.5)	2.94 (1.72‒5.03)	<0.001
Acquisition of infection					
Hospital acquired	354 (77.6)	101 (66.4)	253 (83.2)	0.40 (0.25‒0.63)	<0.001
Community acquired	102 (22.4)	51 (33.6)	51 (16.8)	2.50 (1.60‒3.93)	<0.001
Clinical diagnosis					
*Clostridium*-associated diarrhea	375 (82.2)	96 (63.2)	279 (91.8)	0.15 (0.09‒0.26)	<0.001
Extraintestinal clostridial infection	81 (17.8)	56 (36.8)	25 (8.2)	6.51 (3.85‒11.01)	<0.001
Bacteremia	8 (1.8)	7 (4.6)	1 (0.3)	14.63 (1.78‒120.00)	0.012
Intra-abdominal infection	31 (6.8)	21 (13.8)	10 (3.2)	4.71 (2.16‒10.23)	<0.001
Biliary tract infection	4 (0.9)	3 (2.0)	1 (0.3)	6.10 (0.63‒59.15)	0.119
Recurrent infection	15 (3.3)	0 (0)	15 (4.9)	NA	NA
Skin and soft tissue infection	36 (7.9)	23 (15.1)	13 (4.3)	3.99 (1.96‒8.13)	<0.001
Genital tract infection§	2 (0.4)	2 (1.3)	0 (0)	NA	NA
Complication					
Ileus	34 (7.5)	17 (11.2)	17 (5.6)	2.12 (1.05‒4.29)	0.035
Bowel perforation	14 (3.0)	11 (7.2)	3 (1.0)	7.83 (2.15‒28.50)	0.002
Hypovolemic or septic shock	43 (9.4)	22 (14.5)	21 (6.9)	2.28 (1.21‒4.30)	0.011
Mortality					
30-day mortality	81 (17.7)	22 (14.5)	59 (19.4)	0.70 (0.41‒1.20)	0.195
90-day mortality	97 (21.3)	24 (15.8)	73 (24.0)	0.59 (0.36‒0.99)	0.045
Overall mortality	122 (26.7)	35 (23.0)	87 (28.6)	0.77 (0.49‒1.21)	0.264

*Values are no (%) except as indicated. p value of ORs was analyzed by univariate logistic regression. ICU, intensive care unit; IQR, interquartile range; NA, not applicable for continuous variables or too few events (<5) to calculate a stable OR; OR, odds ratio. †By independent *t* test. ‡By Mann-Whitney test. §Two cases were diagnosed as pyospermia and bacterial vaginitis.

### Disease Characteristics and Severity

We observed notable differences in disease characteristics between the 2 groups. Those in the CI group had a 6.5 times higher risk of developing EICI, including bacteremia, intra-abdominal infection, biliary tract infection, skin and soft tissue infection, pyospermia, and bacterial vaginitis (36.8% for CI vs. 8.2% for CD; OR 6.5, 95% CI 3.9 –11.0; p<0.001) (Table 1). On the contrary, most disease manifestation in the CD group was confined to the intestine and colon, mainly *C. difficile*–associated diarrhea. Most patient had antibiotic exposure 30 days before the CI or CD infection event. CD group showed higher 30-day antibiotic exposure rate (95.1%) than CI group (79.6%; p<0.001) (Table 2) and longer duration (mean 15.6 days, SD 8.3) than CI group (mean 13.7 days, SD 8.6; p<0.001). Patients in CD group received more penicillins, cephalosporins, carbapenems, and fluroquinolones (Table 2).

**Table 2:** Antibiotic exposure before *Clostridium* infection in case–control study of *C. innocuum* infection *

Antibiotic exposure	*C. innocuum*, n = 152	*Clostridioides difficile*, n = 304	Odds ratio (95% CI)	p value
Any antibiotic exposure	121 (79.6)	289 (95.1)	0.20 (0.11–0.39)	<0.001
Mean duration of antibiotic exposure, d (SD)†	13.7 (8.6)	15.6 (8.3)	NA	0.039
Antibiotic exposure rate by drug class				
Penicillins	36 (23.7)	107 (35.2)	0.57 (0.37–0.89)	0.013
Cephalosporins	81 (53.3)	206 (67.8)	0.54 (0.36–0.81)	0.003
Carbapenems	36 (23.7)	102 (33.6)	0.62 (0.40–0.96)	0.031
Fluoroquinolones	31 (20.4)	108 (35.5)	0.47 (0.29–0.74)	0.001
Aminoglycosides	13 (8.6)	20 (6.6)	1.33 (0.64–2.75)	0.444
Macrolides	3 (2.0)	8 (2.6)	0.75 (0.20–2.85)	0.667
Tetracyclines	6 (3.9)	5 (1.6)	2.46 (0.74–8.19)	0.143
Glycopeptides	52 (34.2)	92 (30.3)	1.20 (0.79–1.81)	0.393
Oxazolids	0 (0)	2 (0.7)	NA	NA
Polymyxins	6 (3.9)	11 (3.6)	1.10 (0.40–3.02)	0.861
Lincosamides	6 (13.9)	21 (6.9)	0.55 (0.22–1.40)	0.213
Metronidazole	16 (10.5)	33 (10.9)	0.97 (0.51–1.81)	0.915

*Data are presented as no (%) unless otherwise indicated. NA, not applicable for continuous variables or too few events (<5) to calculate a stable odds ratio. †By independent student *t* test. The p value of odds ratio was analyzed by univariate logistic regression.

Regarding disease severity, most of the patients in both groups required hospitalization (93.4% in the CI group and 97.7% in the CD group; p = 0.03) (Table 1). Although most patients in CD group had intestinal infections, gastrointestinal tract–related complications of ileus, bowel perforation, clinical sepsis, and shock occurred more frequently in the CI group (26.3%) than CD group (11.2%; OR 2.8, 95% CI 1.7–4.7; p<0.001). CI group also showed a higher rate of intensive care unit (ICU) admission (23.6% vs. 9.5%; OR 2.9, 95% CI 1.7–5.0; p<0.001) (Table 1). All the data indicated that the disease severity at the acute stage was more severe and invasive in the *C. innocuum*–infected patients. Furthermore, we saw no recurrence of infection in CI group but recurrence of infection in 4.9% of CD group (p = 0.005).

We observed no statistically significant differences in clinical presentations, but patients with *C. innocuum* infection had fewer diarrheal symptoms and less fever. In the laboratory testing results, patients experienced anemia more commonly in the CD group than CI group; hemoglobin counts were 9.8 (2.0) g/dL in CD and 10.7 (2.4) g/dL in CI (p<0.001) (Table 3). We observed no difference in other systemic inflammatory markers. A limited number of patients received colonoscopy examination, and we found no pseudomembranous colitis in the CI group.

**Table 3:** Clinical and laboratory characteristics by the infecting *Clostridium* species in case–control study of *C. innocuum* infection, Taiwan*

Characteristic	*C. innocuum*, n = 152	*Clostridioides difficile*, n = 304	p value
Clinical symptoms			
Diarrhea	56 (36.8)	217 (71.4)	<0.001
Fever	29 (19.1)	92 (30.3)	0.011
Abdominal pain	37 (24.3)	54 (17.8)	0.098
Vomiting	13 (8.6)	29 (9.5)	0.731
Abdominal distension	25 (16.4)	41 (13.5)	0.391
Blood testing			
Leukocytes, cells/μL†	10,454 (6,773)	11,005 (6,788)	0.783
Hemoglobin, g/dL†	10.7 (2.4)	9.8 (2.0)	<0.001
Platelet count × 1,000/μL†	243 (110.4)	231 (135.0)	0.134
CRP, mg/L, median (IQR)‡	55.7 (104.7)	55.7 (97.2)	0.108
Stool routine, no. positive/total (%)			
Occult blood	53/73 (72.6)	175/216 (81.0)	0.128
Mucus	9/70 (12.8)	39/205 (19.0)	0.241
Pus cells	8/70 (11.4)	30/205 (14.6)	0.502
Sample site			
Stool	96 (63.2)	279 (91.8)	<0.001
Blood	7 (4.6)	1 (0.3)	0.001
Ascites	13 (8.5)	8 (2.7)	0.002
Bile§	2 (1.3)	1 (0.3)	0.219
Pus/abscess§	16 (10.5)	3 (1.0)	<0.001
Wound/deep tissue§	16 (10.5)	12 (3.6)	0.006
Endocervix§	1 (0.7)	0	0.592
Semen§	1 (0.7)	0	0.592
Antimicrobial susceptibility#			
Metronidazole	20/20 (100)	53/53 (100)	1.000
Clindamycin§	30/44 (68.2)	17/20 (85.0)	0.158
Penicillin§	35/44 (79.5)	12/20 (60.0)	0.101
Ampicillin/sulbactam	21/21 (100)	44/44 (100)	1.000

*Values are no. (%) patients except as indicated. Among patients with *C*. *difficile* (CD)–associated diarrhea, the CD toxin assay positive rate was 65%. χ^2^ tests were used to compare all the categorical variables listed in the table except as noted. CRP, C-reactive protein; IQR, interquartile range. †By independent *t* test. ‡By Mann-Whitney test. §By Fisher exact test. #Data are expressed as susceptible isolate number/total isolate number (%).

### Outcome and Risk Factor for Mortality Rate 

The 30-day mortality rate in the CI group was 14.5%; the 90-day rate, 15.8%, and the overall rate, 23.0%. Although the 90-day mortality rate was slightly higher in the CD group with a significant difference (p value of log rank test = 0.05) in Kaplan-Meier survival analysis, the overall mortality rate did not show a statistically significant difference between the 2 groups (Figure). Using logistic regression, we identified chronic kidney disease (OR 8.6, 95% CI 2.6–28.4; p<0.001), solid tumor (OR 3.5, 95% CI 1.0–12.0; p = 0.051), ICU admission (OR 7.3, 95% CI 2.4–21.9; p<0.001), and shock status (odds ratio 8.0, 95% CI 2.4–27.2; p<0.001) as 4 independent risk factors for both 30-day and overall mortality rates in the patients with *C. innocuum* infection. We identified 7 bacteremias caused by *C. innocuum* in this study. Two of those patients experienced septic shock, and 1 needed ICU hospitalization. The 30-day mortality rate for the 7 patients was 42.9% (3/7) and 90-day was 57.1% (4/7).

**Figure:**
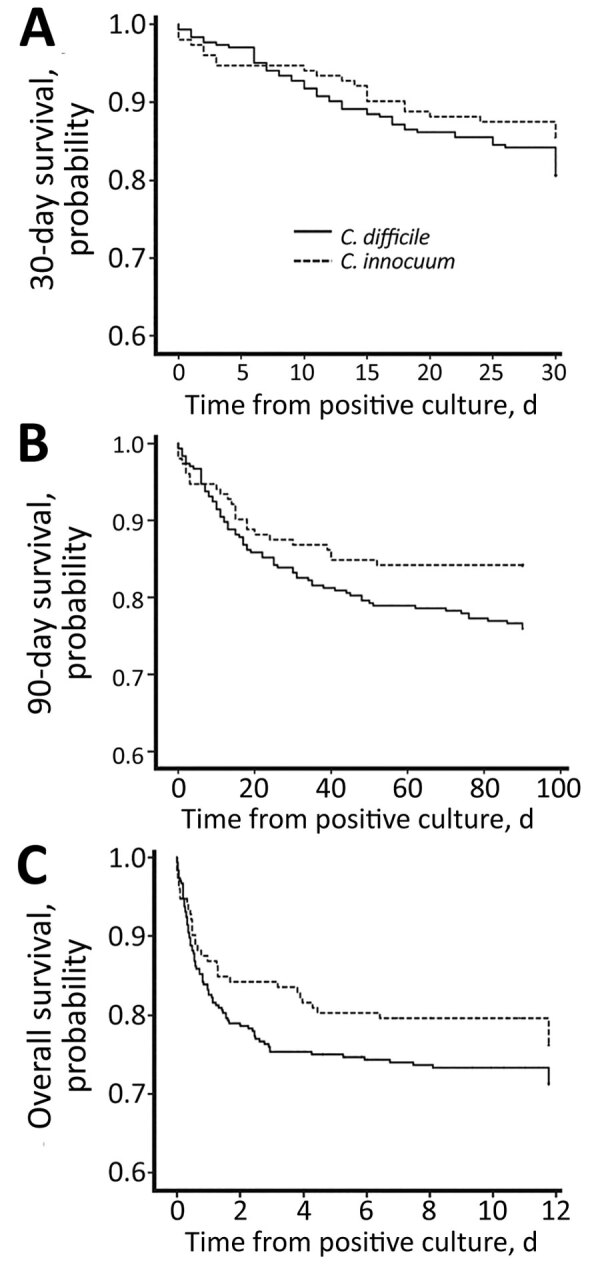
Kaplan-Meier curve of 30-day (A), 90-day (B), and overall (C) survival rates of patients with *Clostridioides difficile* and *Clostridium innocuum*, Taiwan. In the *C. innocuum* group, the 30-day survival rate was 85.5%, 90-day survival rate 84.2%, and overall survival rate 77.0%. The 90-day survival rate was slightly higher than the *C. difficile group* (p value of log rank test = 0.05), whereas the 30-day and overall survival rates did not show a significant difference between the 2 groups.

### Microbiologic Result and Antimicrobial Susceptibility

Among the 152 *C. innocuum* isolates, we recovered 96 (63.2%) isolates from stool specimens; the rest were from the blood (*7*), ascites (*13*), pus/abscess (*16*), wound/deep tissue (*16*), bile juice (*2*), endocervix (*1*), and semen (*1*). We detected 18 polymicrobial infections in the CI group, most of which were from ascites and pus/abscess samples. More *C. innocuum* isolates (36.8%) than *C. difficile* isolates (8.2%) were from extraintestinal specimens (p<0.001) (Table 3), which is compatible with our clinical observation. We performed antimicrobial susceptibility testing on limited isolates. In the *C. innocuum* isolates, we observed the highest susceptibility rate for metronidazole (20/20, 100%) and ampicillin/sulbactam (21/21, 100%), followed by penicillin (35/44, 79.5%) and clindamycin 30/44 (68.2%).

## Discussion

Genus *Clostridium* is large and heterogeneous; it includes <200 species. Accurate species identification has been difficult. In recent years, several new species have been recognized and others reclassified using newer molecular diagnostic methods, such as 16S rRNA gene sequencing (*24*). Among the medically important *Clostridium* spp., *C. perfringens* is the predominant species isolated from cases of bacteremia. The severity of EICI varies; for bacteremia, the mortality rate was found to be 48%–52% by different studies (*25*–*27*). The risk factors for disease acquisition and death were related to an underlying immunocompromised condition such as hemodialysis, malignancy, immunosuppressant use, and Crohn’s disease (*25*). The main portal of entry is the hepatobiliary and gastrointestinal tract. We believe this is also the case in *C. innocuum* because stool was a common source for the *C. innocuum* isolates and gastrointestinal tract–related complications were not uncommon in *C. innocuum*–infected patients. A recent study by Ha et al. (*28*) also found that *C. innocuum* is one of the most common bacteria that could translocate from intestine to mesenteric tissue in patients with Crohn’s disease and further induce adipogenesis and local fibrosis, known together as creeping fat.

We found that among anaerobic clostridial species, *C. innocuum* has long been overlooked as a human pathogen. Our study is to date the most comprehensive observational study to depict the clinical manifestations and outcome of *C. innocuum* infection; not only it is more invasive than most *Clostridium* species, but it can cause more gastrointestinal tract complications following intestinal infection. Case reports of EICI related to *C. innocuum* infection have been published from the United States, Spain, Japan, and Taiwan (*10*,*29*–*32*) (Table 4). Bacteremia and intra-abdominal infection were the most common manifestations, which is compatible with our observations. All the infections occurred in patients with underlying conditions; prolonged antimicrobial therapy was required to treat these patients, whose mortality rate (20%) was similar to that observed in our study (23%). Compared to *C. difficile*, which is known to be a nosocomial pathogen, nearly one third of the *C. innocuum* infections occurred in the community. This observation indicates that *C. innocuum* could be more virulent and competitive than *C. difficile*.

**Table 4:** Reported cases of extraintestinal *Clostridium innocuum* infection, 2000–2020, Taiwan*

Characteristic	Castiglioni et al. (*10*)	Crum-Cianflone et al. (*29*)	Hung et al. (*30*)	Mutoh et al. (*31*)	Aroca-Ferri et al. (*32*)
Year and country	2003 United States	2009 United States	2014 Taiwan	2015 Japan	2019 Spain
Age, y/sex	38/F	38/M	85/M	32/M	44/F
Underlying conditions	Chronic HCV, interstitial nephritis after renal transplant	AIDS	DM with CDAD and CMV colitis	ALL	Takayasu arteritis, ESRD under PD
Isolation site	Blood	Blood	Blood	Blood, BM	Peritoneal fluid
Vancomycin MIC	16 μg/mL	NA	>32 μg/mL	8 μg/mL	8 μg/mL
Diagnosis	Bacteremia secondary to infectious hematoma	Bacteremia	Bacteremia	Pelvic osteomyelitis complicated with iliac muscle abscess	PD peritonitis complicated with sigmoid colon perforation
Treatment	IV TZP, IV CLI	IV DAP, PO MTZ	IV TZP	IV TZP, IV MTZ, IV CLI	IV CTX, IP AMP, IP CLI
Duration	11 days and surgery	NA	2 weeks	8 weeks	15 days
Outcome	Recovered	Recovered	Recovered	Recovered	Died

*ALL, acute lymphoblastic leukemia; AMP, ampicillin; BM, bone marrow; CDAD, *C. difficile*–associated diarrhea; CLI, clindamycin; CMV, cytomegalovirus; CTX, cefotaxime; DAP, daptomycin; ERY, erythromycin; ESRD, end stage renal disease; HCV, hepatitis C virus; IP, intraperitoneal route; IV, intravenous route; MIC, minimum inhibitory concentration; MTZ, metronidazole; NA, not available; PD, peritoneal dialysis; PO, oral route; TZP, piperacillin/tazobactam.

Among the EICI, bacteremia is the most severe form of infection. In a recent study by Morel et al. (*33*), non–*C. difficile* *Clostridium* bacteremia requiring ICU hospitalization showed an aggressive clinical course and was usually life-threatening. The 28-day mortality rate was 55% and the 90-day mortality rate was 71% (*33*). This report is compatible with our findings of 30-day (42.9%) and 90-day (57.1%) mortality rates in the CI bacteremic patients.

Identifying *C. innocuum* infection is important because the microorganism expresses intrinsic resistance to vancomycin, because of the synthesis of peptidoglycan precursors with low affinity for vancomycin (MIC 4–16 mg/L) (*8*,*26*). Moreover, highly vancomycin-resistant strains (MIC >16 mg/L) could develop if the bacteria were previously exposed to vancomycin (*34*). Because oral vancomycin has been recommended as the first-line therapy for *C. difficile* infection, distinguishing *C. innocuum* from other clostridial species becomes essential to avoid treatment failure caused by inappropriate antimicrobial use. Metronidazole and clindamycin appear to be appropriate choices for treating *C. innocuum* infection, according to our antimicrobial susceptibility testing results.

The main limitation of our study is the retrospective study design and the inevitable missing data. The lack of standardized medical record format prevented us from precisely defining every case-patient’s diagnosis, especially antibiotic-associated diarrhea and acute colitis, which have similar clinical descriptions in the medical records. Some objective data were not available, which may potentially compromise the accuracy of the estimated rates of presentations and diagnoses among the patients. However, the proportion of missing data appeared small and should not significantly affect the results of the study. Second, not all the *C. innocuum* isolates from the enrolled patients were tested for antimicrobial susceptibility, and that testing did not include vancomycin. Third, the study does not advance our understanding on virulence mechanism of *C. innocuum*. It is possible that *C. innocuum* possesses a unique virulence mechanism to cause gastrointestinal as well as extraintestinal infections, such as the lipopolysaccharide-like structure we described in our previous study (*9*). *C. difficile* also contains surface lipocarbohydrate, which has a similar biologic activity to the lipopolysaccharide in gram-negative bacteria (*35*); this hypothesis needs further experimental verification.

In conclusion, *C. innocuum* should be considered an important *Clostridium* species causing EICI and gastrointestinal infection that has a risk for severe complications and a high mortality rate in immunocompromised patients; physicians should recognize it as a pathogen to treat clinically. More studies are needed to understand the virulence mechanism of *C. innocuum*. Precise identification of *C. innocuum* will guide appropriate and timely antimicrobial therapy for patients because of its intrinsic vancomycin resistance.
